# The Making and Evaluation of Digital Games Used for the Assessment of Attention: Systematic Review

**DOI:** 10.2196/26449

**Published:** 2021-08-09

**Authors:** Katelyn Wiley, Raquel Robinson, Regan L Mandryk

**Affiliations:** 1 Department of Computer Science University of Saskatchewan Saskatoon, SK Canada

**Keywords:** cognitive assessment, attention, serious games, gamification, systematic review, mobile phone

## Abstract

**Background:**

Serious games are now widely used in many contexts, including psychological research and clinical use. One area of growing interest is that of cognitive assessment, which seeks to measure different cognitive functions such as memory, attention, and perception. Measuring these functions at both the population and individual levels can inform research and indicate health issues. Attention is an important function to assess, as an accurate measure of attention can help diagnose many common disorders, such as attention-deficit/hyperactivity disorder and dementia. However, using games to assess attention poses unique problems, as games inherently manipulate attention through elements such as sound effects, graphics, and rewards, and research on adding game elements to assessments (ie, gamification) has shown mixed results. The process for developing cognitive tasks is robust, with high psychometric standards that must be met before these tasks are used for assessment. Although games offer more diverse approaches for assessment, there is no standard for how they should be developed or evaluated.

**Objective:**

To better understand the field and provide guidance to interdisciplinary researchers, we aim to answer the question: How are digital games used for the cognitive assessment of attention made and measured?

**Methods:**

We searched several databases for papers that described a digital game used to assess attention that could be deployed remotely without specialized hardware. We used Rayyan, a systematic review software, to screen the records before conducting a systematic review.

**Results:**

The initial database search returned 49,365 papers. Our screening process resulted in a total of 74 papers that used a digital game to measure cognitive functions related to attention. Across the studies in our review, we found three approaches to making assessment games: gamifying cognitive tasks, creating custom games based on theories of cognition, and exploring potential assessment properties of commercial games. With regard to measuring the assessment properties of these games (eg, how accurately they assess attention), we found three approaches: comparison to a traditional cognitive task, comparison to a clinical diagnosis, and comparison to knowledge of cognition; however, most studies in our review did not evaluate the game’s properties (eg, if participants enjoyed the game).

**Conclusions:**

Our review provides an overview of how games used for the assessment of attention are developed and evaluated. We further identified three barriers to advancing the field: reliance on assumptions, lack of evaluation, and lack of integration and standardization. We then recommend the best practices to address these barriers. Our review can act as a resource to help guide the field toward more standardized approaches and rigorous evaluation required for the widespread adoption of assessment games.

## Introduction

### Attention

From crossing the street to composing a tweet, functioning as a human always requires people to take in information, process it, and respond accordingly. Whether in the lab or in the world, detecting stimuli and responding to them, both consciously and unconsciously, involves many cognitive functions. One of these important cognitive functions is *attention*, which Kahneman describes as “a label for some of the internal mechanisms that determine the significance of stimuli” [1].

These *internal mechanisms* of attention can be divided into multiple types. Common areas of attention include selective attention (how people attend to relevant information and ignore irrelevant information), divided attention (when people attend to multiple things at once), and sustained attention (the ability to focus on something for a continuous amount of time) [2,3].

There are also models of attentional control that describe the difference between involuntary and voluntary attention. Attentional control is related to inhibition, shifting, and updating. Inhibition involves preventing irrelevant stimuli from impairing performance, shifting refers to the allocation of attention to whatever is most relevant at the time, and updating is how people encode new information into working memory [4].

### Assessment of Attention

Measuring and understanding attention and attentional control are important, as attention is a major cognitive function that influences human development and mental health. Furthermore, as attention is related to a variety of cognitive deficits (eg, attention-deficit/hyperactivity disorder [ADHD] [5] and dementia [6]) and abilities (eg, reading [7]), an accurate measure of attention can help assess and diagnose a number of common disorders.

Measuring attention, and other aspects of cognition such as memory and perception, is often done using *cognitive tasks*. A common approach is to present participants with stimuli and ask them to respond in different ways, while measuring their reaction time and accuracy (ie, how quickly they attend to stimuli and if they respond in the way intended). Research on attention has often relied on specific cognitive tasks, such as the Eriksen flanker task [8] and the Posner cueing task [9], which have been fundamental to the study of attention. More recent cognitive tasks continue to advance the knowledge of attention. For example, the dot probe task demonstrates that people with anxiety preferentially attend to threatening stimuli [10], and attentional blink tasks support the idea that attentional resources are limited [11].

Cognitive assessment tasks have specific standards that must be met before they are widely used, especially in clinical settings. They are expected to have certain psychometric properties, such as validity (how well they measure what they claim to measure), reliability (how consistent the test is), sensitivity (how well they identify true positives), and specificity (how well they identify true negatives).

### Digital Games for the Assessment of Attention

Although cognitive assessment tasks are standardized and highly used, they do have some limitations. They can be expensive, as many require trained experts to administer the tasks [12]. They are boring and repetitive, which can cause difficulties with recruitment for research and with patient cooperation for clinical use [13]. The data collected by these tasks can be unreliable, as participants might not be fully engaged and often exert suboptimal effort [14,15]. They also lack ecological validity [12,16]; therefore, they may not be indicative of how these cognitive skills affect daily functioning. To address these limitations, researchers have started to integrate elements from computer and video games into cognitive tasks for assessment.

Games have the potential to improve the quality and quantity of collected data by increasing participants’ engagement in the moment (better data) and by engaging many more people over longer periods (more data) [17]. For example, using a game called *Sea Hero Quest* [18], researchers were able to collect spatial navigation data from over 4.3 million people, which would be near impossible with a traditional paper-based task or even a standard digitized assessment. However, although assessment games can be very successful, they do not always improve participant enjoyment. Vanden Abeele et al [19] noted the importance of game *quality* when developing assessment games. In fact, studies have shown that some game elements are associated with lowered enjoyment compared with traditional tasks [13,17].

Game elements can also hinder the assessment properties of a task. Cognition is complicated, and traditional tasks are heavily studied before researchers can be confident that they measure what they claim to measure in a consistent way (issues of validity and reliability). Even a small change in a task must be studied to understand its effects [20].

The use of games to measure cognitive processes related to attention poses unique issues. Through their use of graphics, stimuli, and visual feedback, games inherently manipulate the player’s attention, which has been shown to be problematic when using games for assessment. For example, Wiley et al [17] found that participants responded more quickly but also less accurately to a dot probe task when points were awarded for faster, correct responses. Other features could also manipulate attention; for example, increasing narrative suspense has been linked to a narrowed attentional focus [21]. In go/no-go games, using *gamelike stimuli* such as cartoon characters has resulted in decreased performance compared with standard tasks, possibly because it is more difficult to differentiate between complicated graphical stimuli than simple colored shapes [13].

Attention can also interact with games based on individual differences. A study by Delisle and Braun [22] found that game elements can normalize the performance of individuals with ADHD. They designed a task to resemble a fast-paced video game and found that the presence of game elements improved the performance of participants with ADHD more than that of non-ADHD participants. This unequal effect on performance implies that a game designed to assess ADHD could, ironically, be rendered unable to discriminate effectively. Other individual differences may also affect the data, such as differences in age, gender, and game-playing experience. Studies have shown that action video game players demonstrate visual search advantages [23]; such a difference may be emphasized by using a game to measure attention. Similarly, older adults or people with little gaming experience may perform poorly on a game, not because they have lower attention abilities but because they are less familiar with computers and games.

### This Research

There has been considerable research interest in the use of gamification (ie, the use of game elements in nongame contexts [24]) on cognitive tasks, which has been synthesized in several review papers [25,26]. However, the focus of much of this synthesis research has been on the gamification of *training* and *intervention* [26], with less systematic exploration of the efficacy of games for *assessment*. Although games for training and assessment are often grouped together, recent research suggests that gamification may not be the best approach for assessment [17]. Although cognitive tasks are standardized and have been heavily researched, serious games for assessment are diverse, and there is no field-wide standard for how they should be developed. Our systematic review seeks to explore the different approaches to using games for assessment, particularly for assessing attention, which can be complex. In comparison to other systematic reviews, we provide two unique contributions: First, we look beyond gamification to other approaches for developing assessment games. Second, we review the methodology of developing and evaluating serious games for assessment, rather than just the end product. We aim to provide a guide for interdisciplinary researchers on the development and evaluation of assessment games. Our main research question is: How are digital games used for the cognitive assessment of attention made and measured?

## Methods

### Eligibility Criteria

Our eligibility criteria required each included paper to be published before March 1, 2021, a peer-reviewed journal article or conference proceeding, primary research (ie, not a literature review or background article), and written in English.

**Figure 1:**
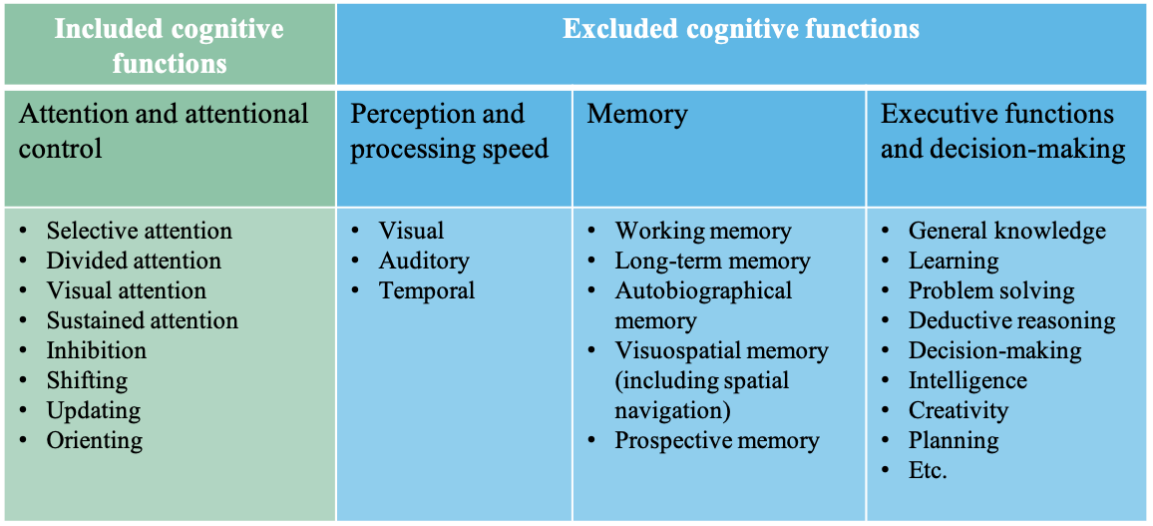
Included and excluded cognitive functions for the eligibility criteria in our review.

In addition, each paper needed to include a *digital game* used for the *assessment of attention-related processes* that could be used *remotely*. For this criterion, we used the following definitions:

- Digital game: As there are many ways to define a *game*, we chose to follow the original researchers’ intentions. If the authors of a paper referred to an assessment as a *game*, *gamified*, or some other variation, we included the paper.
- Attention: We included papers related to attention and attentional control. Figure 1 presents the detailed list of the cognitive processes included.
- Assessment: We were interested in studies that sought to measure attention, for purposes of either detection or diagnosis, research, or monitoring cognitive changes. Studies focused on treatment, training, or interventions were excluded, as were studies on educational and work assessments (eg, assessing for employee selection or how well a concept was learned).
- Remote: As the goal of digitizing assessment is often to increase its scale, accessibility, and reach [27], we included only papers where the game could potentially be deployed remotely, using only a computer, tablet, or phone. Studies that required specialized hardware (eg, a Microsoft Kinect, gaming controllers, and any custom hardware) were excluded, although studies with commonly used devices (eg, a mouse, headphones, and keyboard) were included. Though the ability for studies to be deployed remotely depends on more than available hardware, for this review we did not exclude studies without the requisite software.

### Information Sources and Search Strategy

We searched the titles and abstracts of papers across several databases, chosen for their relevance to games user research and psychology: ACM Digital Library, IEEE Xplore, PubMed, PsycINFO, Scopus, and Web of Science. Our keywords were used in a search string adapted to the requirements of each database but generally followed the same format. In addition, where possible, we added the requirement that the returned records be articles published in English. We set no lower time limit but did require that the included papers be published before March 1, 2021. To facilitate other potential systematic reviews, we cast a wide net with our search terms and included other cognitive processes. For example, for Scopus, the search strings were as follows: TITLE-ABS ((gamif* OR game OR games) AND (cognit* OR neuropsych* OR assessment OR memory OR executive function OR attention* OR impulse control OR processing speed OR inhibition OR anxiety OR depression)) AND DOCTYPE (ar).

### Study Records and Data Management

To screen the final set of records for our inclusion criteria, we used Rayyan, a web-based system developed for conducting systematic reviews [28].

The first author screened the titles and abstracts of all records for obvious exclusions, such as papers that referenced physical games and sports, as opposed to digital games. The first 2 authors screened the remaining papers, made final decisions, and resolved conflicts through discussion.

As a final quality check, we also screened the first 100 results from Google Scholar using the search string: ((gamif* OR game OR games) AND (attention*)).

### Data Items and Synthesis

Our main research question for this systematic review is: How are digital games used for the cognitive assessment of attention made and measured? We also had follow-up questions related to the evaluation of the game’s efficacy and engagement: how effective are the games for accurate assessment? and how effective are they in terms of participant engagement?

To conduct the review, we gathered data related to a list of questions for each paper using a spreadsheet. We listed the specific area of attentional control the study focused on (eg, selective and divided), the population examined (eg, children and older adults), and the sample size of the study.

We also listed a number of details about each assessment, including its intended purpose, a general description of the assessment, and if it was focused on a specific disorder (eg, ADHD, dyslexia, or dementia). We were also interested in how each assessment was measured, particularly how it was evaluated, if it was compared with any traditional cognitive tasks or a clinical diagnosis, and any results from the evaluation.

We listed details about gameplay, giving a general description of each game and listing any game mechanics used (eg, points, narrative, and avatars). We also noted how the game was developed (eg, gamification of a task, custom game, and existing commercial game), the expertise of individuals involved in its development (eg, health care professionals and game designers), any evaluation of the game and the results (eg, enjoyment and immersion), and the authors’ motivation for using a game.

The first 2 authors collected the data from each paper in a spreadsheet, with each author responsible for half of the papers. The first author then reviewed each paper and the spreadsheet to ensure uniform data collection. These data then informed the qualitative synthesis presented in our results. The wide range of methods used in the included papers precluded any meta-analysis or meaningful quantitative analysis. At most, we provided the summary statistics. We intended for this review to provide an overview of how research is conducted in the field and focus on the methodology of each paper.

## Results

### Search Results

Our initial search was conducted in December 2019, with an updated search conducted in March 2021 to include any new publications. The two searches resulted in a set of 91,968 records. We used Mendeley Reference Management software, which automatically deleted 38,179 duplicate records. We then manually deleted remaining 4424 duplicates, resulting in a final set of 49,365 records to review.

The first author’s initial screening excluded 46,969 records. These exclusions were made quickly based on brief searches through titles and abstracts. For example, many records referenced *the Olympic Games* or *game* as in animal game.

The first 2 authors then reviewed the remaining 2396 papers in more detail and excluded a further 2326 papers. At this stage, common exclusions included papers that addressed cognitive processes other than attention [29], papers that used virtual reality or other specialized hardware [30], and papers that focused on interventions and training [31].

**Figure 2:**
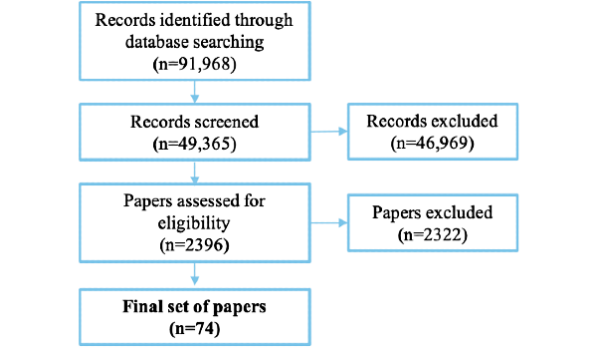
Flowchart of included and excluded records in our review.

A total of 78 papers were selected for analysis; however, we were unable to obtain the full text of 4 papers from any digital library, interlibrary loan, or attempting contact with the authors, leaving a final set of 74 papers for the review (Figure 2).

### Summary of Included Studies

The full details of the papers included in this review can be found in Multimedia Appendix 1 [13,17,32-103] (general details of each study) and Multimedia Appendix 2 [13,17,32-103] (assessment and game details of each study).

#### Publishing Formats and Dates

The 74 papers in our review were published in journals from a variety of fields (based on the journal descriptions). Of these 74 papers, 33 (45%) papers were from psychology and medical publications, 26 (35%) papers were from interdisciplinary publications, 13 (18%) papers were from computer science publications, and 2 (3%) were from education publications (Table 1).

**Figure 3:**
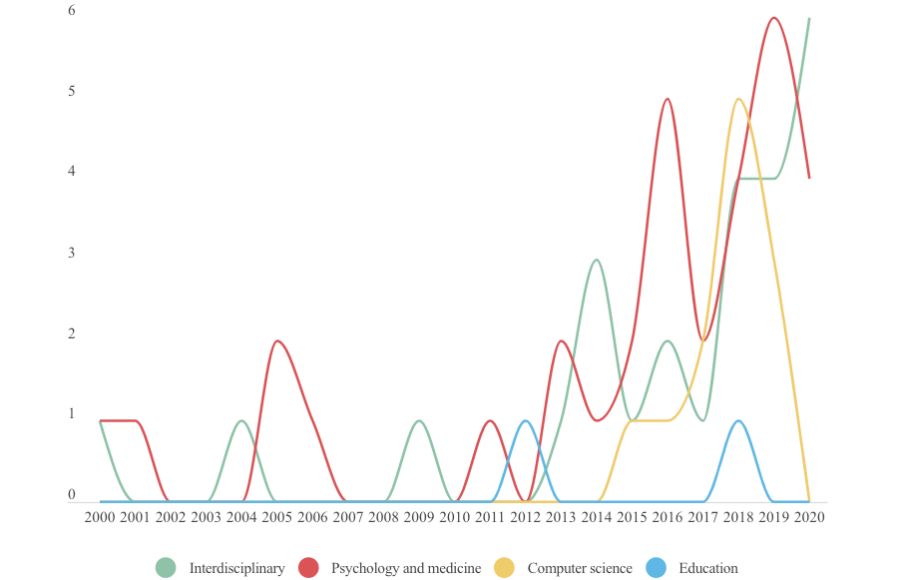
Number of included papers according to publication year and field.

The earliest study in our review was from 2000. Most papers were published in the late 2010s, with 14 papers published in 2018 and 13 in 2019 (Figure 3). Two papers were published in the first 2 months of 2021, before the upper time limit for inclusion in this review.

**Table 1:** References to all included papers according to publication field.

Field	Reference
Interdisciplinary	[13,17,32-55]
Psychology and medicine	[56-88]
Computer science	[89-101]
Education	[102,103]

#### Study and Participant Characteristics

The selected papers included 40,154 participants, with sample sizes ranging from 5 to 16,233 participants.

The majority of studies (35) focused on children, with 12 papers focusing on older adults and 25 on a general adult population. In addition, 2 papers had participants from across the human life span, with both children and adults of all ages.

Although the majority of papers (34) looked at a general population, 3 main disorders were also studied: 16 papers on dementia and general cognitive impairment, 11 on ADHD, and 7 on dyslexia. Furthermore, 6 papers examined other disorders (ie, schizophrenia, substance abuse, aggression, multiple sclerosis, Down syndrome, Zika virus disease, Parkinson disease, and Huntington disease).

The papers studied many different aspects of attention and attentional control: inhibition (25 papers), sustained attention (23 papers), visual attention (19 papers), selective attention (14 papers), switching (9 papers), updating (7 papers), divided attention (6 papers), orienting (2 papers), and attentional bias (1 paper). Furthermore, 23 papers measured multiple types of attention. In addition, 5 papers did not specify what type of attention was measured nor could it be inferred from the information in the papers.

### Why Are Digital Games Used for Assessment?

The papers in our review listed several reasons for using a game to assess cognition: to address the limitations of traditional tests (32 papers), to increase participant motivation (22 papers), to engage children (18 papers), and because of previous research (7 papers). In addition, 7 papers listed multiple reasons, whereas 7 papers did not list a motivation for using a game.

The most common reason for using a game was to improve motivation and engagement (22/40, 55% with a general population; 18/40, 45% specifically geared toward children). For example, Thirkettle et al [38] created an app that gamified a battery of cognitive tests, specifically with the goal of encouraging repeated play. Dibbets et al [85] sought to study task switching in children but realized that traditional tasks require participants to be literate. Thus, they developed the Switch Task for Children, which does not require a reading response and presented it as a game to “appeal to young children.”

In total, 32 papers used games to address the limitations of traditional assessments, as they can be costly in terms of time and resources, require special expertise, lack ecological validity, and cannot be widely deployed. For example, Brown et al [49] collected data from over 16,000 users using a smartphone app that gamified several tasks. Tong et al [70] created a *whack-a-mole* game for delirium screening in emergency departments, noting that it would be particularly useful to have an automated cognitive test given the busy and demanding nature of emergency rooms.

### How Are Digital Games Used for Assessment Made?

#### Overview

Our review found three different approaches to developing games for assessment purposes: gamifying cognitive tasks (33 papers), creating custom games based on theories of cognition (37 papers), and exploring potential assessment properties of commercial games (4 papers). One study used both a gamified task and a commercial game.

#### Gamification

Gamifying a traditional cognitive task is a common approach for making a game for assessment. This approach involves adding points, graphics, and other game elements to a traditional task. Typically, these tasks are digitized (if they are not already), and then the game elements are layered over the top of the basic task. As an example of gamification from our review, Johann and Karbach [59] created a series of gamified tasks for children, all based on the story of a magic kingdom where an evil wizard must be defeated. One task was a go/no-go task, where the stimuli were dragons of different shapes and colors. Correct responses advanced a progress bar and earned participants *magic power points*. This example uses points, graphics, and themes to gamify the task, but any one game element or combination of elements can be used for gamification. For example, Lumsden et al [13] created a stop signal gamified task using only points and another variation using only thematic graphics. On the other end of the spectrum, Ryokai et al [36] used many game elements in their multiple object tracking (MOT) game for children, including a theme, graphics, music and other sound effects, dynamically adjusted levels, and feedback.

#### Custom Games

Another popular approach is to create a custom game based on theories of cognition or other previous research. For example, several papers in our review created games to detect dyslexia based on theories of visual-spatial attention [55,89,96,100]. As another example, McKanna et al [32] created *21 Tally*, described as “blackjack played in two dimensions,” based on theories of divided attention.

The custom games in our review are diverse, ranging in appearance and complexity. Some are fairly similar to gamified tasks in their simplicity and approach. For example, although Chesham et al [50] did not gamify a specific task, their custom game looks and feels like a gamified task, with very simple designs and mechanics; in fact, they called it a *taskified game*. Other custom games, such as the *EVO* game by Anguera et al [56], are more akin to a commercial game. *EVO* was designed specifically to resemble a commercial action video game, with a focus on “high-level art, music, feedback and storylines.”

These custom games are also diverse in their development. Some games are very literature- and hypothesis-driven, whereas others offer less rationale for their design. For example, Rauschenberger et al [100] created *MusVis*, a game to detect dyslexia using language-independent methods. They based their design choices on the visual and auditory processing abilities of individuals with dyslexia. On the other hand, some papers in our review did not offer a clear rationale for how they designed their custom game and why they expected it to work.

#### Commercial Games

Four papers used preexisting commercial games. In these cases, the games were not created by the researchers; rather, the researchers explored the potential assessment properties of a game. For example, Intarasirisawat et al [92] used Tetris, Fruit Ninja, and Candy Crush Saga to investigate how touch gestures in popular games might relate to performance on traditional cognitive tasks. In their study, participants were asked to play games and complete traditional paper-based tasks. A bivariate analysis then revealed correlations between commercial games and tasks.

As another example, Houghton et al [52] used Crash Bandicoot to study motor control and sequencing among boys with and without ADHD, under low and high working memory and distractor conditions. The goal of the study was to compare how boys with ADHD performed in an ecologically valid, highly motivating environment (a computer game) compared with a standard laboratory environment.

### How Are Digital Games Used for Assessment Measured?

#### Evaluation of the Assessment Aspect

##### Overview

As digital games have not been traditionally used for cognitive assessments, they need to be evaluated for their relation to cognition; for example, researchers may want to know whether people with and without ADHD display different mouse behaviors [102] or if scores on a gamified go/no-go task correlate to correct responses on a traditional go/no-go task [51,59,62,69].

Our review found three approaches to evaluating the assessment aspect of games: comparison to a traditional task (31 papers), comparison to a clinical diagnosis (14 papers), and comparison to knowledge of cognition (11 papers). An additional 4 papers compared game results with both a traditional task and a clinical diagnosis, and 1 study used both clinical diagnoses and comparisons to normative data. Of these papers, we further identified 4 that used machine learning to evaluate assessment. Thirteen papers did not evaluate the assessment aspect of the game.

##### Comparison to a Traditional Task

The most common approach to evaluating the assessment aspect of a game is to compare the results of a game with the results of an established cognitive task. For example, if the scores from a game are designed to measure response inhibition, researchers may want to compare those scores with those from a go/no-go task. If the patterns of responses are similar, it is likely that the game measures response inhibition in a similar way to a go/no-go task. This process is what Chicchi Giglioli et al [51] followed when evaluating their game, *EXPANSE*, which gamified the dot probe task, go/no-go task, Stroop task, trail making task, and Wisconsin Card Sorting Test. Participants were asked to complete both the standard tasks and game-based versions.

Although this process offers the most direct comparison between tasks and their gamified components, of the 33 papers that gamified a traditional task, only 14 evaluated the game by comparing it with the task.

Custom and commercial games may also be compared with traditional tasks. For example, Tong et al [69] created a *whack-a-mole* game designed to measure response inhibition. Although this game was not based on a specific task, it calculated correlations between performance on the game and performance on a standard Stroop task, which also measures response inhibition. Similarly, Baniqued et al [57] had participants play 20 commercial casual games, as well as a battery of cognitive tasks, and examined the relationships between how participants performed on the games and the tasks.

##### Comparison to a Clinical Diagnosis

Another approach to evaluation is to compare the results of a game with a clinical diagnosis or questionnaire. For example, if a game is designed to measure selective attention, researchers may want to look at how scores on the game differ between children with and without ADHD. As children with and without ADHD typically display different patterns of selective attention, the game can be assessed to determine if it discriminates between children with and without ADHD. If game performance differs between the 2 groups, it may be measuring selective attention. Alternatively, it may be picking up on some other feature of cognition that differs between children with and without ADHD; therefore, this approach needs to be used carefully.

If the intent of the game is to diagnose ADHD, it matters less *why* it works than *if* it works. Most of the games in our review used this evaluation approach for situations in which diagnosis was the goal. For example, Peijnenborgh et al [34] demonstrated that their game, *Timo’s Adventure*, had clinical validity by showing that children with and without ADHD had significant performance differences.

As another example, Fukui et al [88] recruited participants with mild cognitive impairment, participants with Alzheimer disease, and age- and gender-matched healthy control participants and examined whether performance data from their games were able to discriminate between the 3 groups.

##### Comparison With Theories of Cognition

The results of a game can also be compared with the theories of cognition. For example, if a game is designed to examine attentional blink phenomena, we can refer to the literature on patterns of attentional blink to determine if the results from the game make sense. These comparisons can occur in different ways, for example, comparisons with normative data or a specific theory. Brown et al [49] used a set of gamified tasks. They did not compare those games with a task version directly, but they compared their results with the literature on those tasks, reporting that their games produced *canonical results*. Thirkettle et al [38] compared their results on the basis of demographic effects by grouping participant results according to gender and age and assessed the replication of known effects (eg, increases in age correlated with increases in reaction time). Similarly, Ryokai et al [36] compared their results with the known MOT limits.

##### Machine Learning

As an additional finding, we found 4 papers that used machine learning in their evaluation. These studies compared the results of a game with the results of a cognitive task or clinical diagnosis and then used machine learning to build a classification model for the game results. For example, Jung et al [54] used machine learning to classify game scores by comparing them with MMSE scores, and Mwamba et al [33] classified game data based on children with and without a diagnosis of ADHD.

#### Evaluation of the Game Aspect

When using digital games for assessment, it is important to evaluate the games themselves. As discussed earlier, many papers in our review discussed using games as assessment tools with the motivation to increase participant engagement with testing. Thus, researchers may want to know if a game is more enjoyable than a traditional task [62,90] or if participants find the game too difficult to play [101].

In our review, only 25 studies evaluated some aspects of gameplay. The majority of studies (n=41) did not evaluate game features. Furthermore, 8 papers did not formally report an evaluation of the game but suggested that some evaluation was done (eg, a sentence in the discussion section that indicates that most participants enjoyed the game [42] and mentioned that participants were asked if they enjoyed the game without reporting the results [57]).

Of the 25 studies that evaluated game play, most used a short questionnaire to assess enjoyment and difficulty, although these measures vary in complexity. For example, Gaggi et al [89] asked children two simple questions about their game experience: “Do you like the game?” and “Is the game difficult to play?” with simple choices for answers: “Yes, a lot; Yes; Not so much; No” and “Easy; Medium; Hard,” respectively. On the other end of the spectrum, Szalma et al [37] used a more detailed battery of the NASA (National Aeronautics and Space Administration) Task Load Index (a questionnaire that measures perceived workload) and other questionnaires to measure stress, task engagement, and difficulty.

Game behavior was also used as a metric of enjoyment; for example, Thirkettle et al [38] noted that 1400 participants played their game more than 10 times, and Godwin et al [90] found that their *Monster Mischief* game was three times as popular with children as the MOT task it was based on.

Another way to assess game elements is to directly compare multiple versions of a game or task with different elements included in each version. Miranda and Palmer [64] compared three versions of a visual search game: with points and sound, with only points, and with only sound. Lumsden et al [62] used this approach to compare three versions of a gamified task (ie, a nongame task, a gamified task with points, and a gamified task with a theme).

### How Effective Are Assessment Games?

Although our review was mostly concerned with methodology, that is, how assessment games are made and evaluated, we also wanted to explore how effective these games are. How effective are they at an accurate assessment and at participant engagement?

Unfortunately, the general lack of evaluation done by the papers in our review precludes us from answering these questions. In addition, the wide range of methodologies makes it difficult to compare even the limited studies that do include some evaluation. For example, Miranda and Palmer [64] gamified a visual search task and used it to investigate how participants responded to a task with points and sound effects included. Similarly, Lumsden et al [62] used the same approach with a go/no-go task to examine the effects of points and themes. However, the evidence from these studies is difficult to compare despite their similar methodologies. Miranda and Palmer compared versions of their game with only points, with only sound effects, and with both points and sound effects. They did not compare it with the control version of the task. Lumsden et al compared versions of their game with only points, with only theme, and with a control version of the task but did not have a version that combines points and themes. These differences in evaluation were found across all studies in our review.

## Discussion

### Principal Findings

We identified and reviewed 74 papers that used a digital game to measure cognitive functions related to attention. We sought to answer the following question: How are digital games used for the cognitive assessment of attention made and measured?

We found three different approaches to making assessment games: gamifying cognitive tasks, creating custom games based on theories of cognition, and exploring potential assessment properties of commercial games.

Games for assessment have two aspects that can be evaluated: the assessment properties (eg, how accurately attention is measured) and game properties (eg, how fun the game is). The papers in our review that evaluated the assessment properties used three approaches: comparison to a traditional cognitive task, comparison to a clinical diagnosis, and comparison to knowledge of cognition; however, most studies did not evaluate the game properties.

From our review, we identified three barriers to the progress in using games for cognitive assessment. We propose recommendations to address these barriers and offer ideas for further research.

### Barriers to Progress in the Field

#### Overview

There are three barriers to making substantial progress in using games for cognitive assessment. The first barrier we identified is that the literature currently perpetuates assumptions about how users interact with assessment games. Second, there is a lack of evaluation of these games. Third, there is no clear standard for integration across the field.

#### Assumptions

Although the papers in our review did not explicitly state that assumptions about games informed their choices, our results revealed some patterns. In total, 13 papers did not evaluate the assessment aspect of the game. Of these papers, the vast majority were about gamified tasks (9 papers). In addition, of the 33 papers that gamified a traditional task, only 14 evaluated the game by comparing it with the task. This lack of evaluation for gamified tasks may be due to the assumption that adding simple elements such as points and graphics will not interfere with performance on the basic task.

In addition, 40 papers chose to use a game for assessment because of a potential increase in participant engagement and enjoyment; however, this choice was rarely followed up with an evaluation of how enjoyable participants found the game. In fact, of these papers, only 18 evaluated game play. This assumption that a game of any type or quality will be engaging and will yield better results than a traditional task is pervasive across the literature, despite evidence to the contrary. For example, Wiley et al [17] analyzed the effects of including points and theme in a gamified task. They found that although points increased participants’ experiences of enjoyment, challenge, and meaning, adding a theme actually lowered these experiences. After a theme-based introduction to the task, enjoyment was temporarily higher but dropped after play, likely because the basic game play failed to live up to participants’ heightened expectations. Different game experiences influence enjoyment and engagement in ways that cannot always be predicted.

#### Evaluation

More than half (41/74, 55%) of the papers in our review did not evaluate any aspect of game play, and 17% (13/74) of papers did not evaluate any assessment properties. This lack of evaluation is problematic for the advancement of cognitive assessment games. For the games in our review to be seriously considered as assessment tools in the way that standard cognitive tasks are perceived, they need to be evaluated and validated in the same way.

Only 21 papers from our review evaluated both assessment and game properties. As Levy et al [104] noted, “One of the most significant challenges in designing games for scientific studies is the tension between including enough gamelike elements that produce an engaging game, but also selecting the right elements that will not interfere with the validity and reliability of the game as a scientific method or tool.” Each assessment game must be evaluated based on both its assessment value and its game value.

#### Integration and Standardization

The final barrier to progress is integration across the field. Currently, there is little guidance on how assessment games should be made, evaluated, and used. Although every project will be different (eg, each game will use different tasks, themes, or gamification approaches), for the field to advance, there needs to be integration at the level of the game structure. At the structural level, researchers should develop a clear understanding of how different game mechanics in assessment games (eg, points, theme, rewards, feedback, procedures, rules, game input, and narrative elements) interact with user performance and experience. Some research is being conducted in this regard; for example, multiple studies have shown a classic speed-accuracy trade-off when points are included in an assessment game [17,39]. This type of work should be expanded to other game mechanics, and reviews and meta-analyses should integrate the findings to develop standards within the field. The majority of the included studies were published in psychology or medical venues, compared with computer science venues, or interdisciplinary venues; therefore, it could be possible that research teams lack formal training in the design and deconstruction of games [105].

### Recommendations to Address Barriers

#### Overview

To help advance the field, we identified the *best practices* from the papers in our review. These include using clearly defined goals to guide the development of a game, ensuring robust evaluation, and working with interdisciplinary teams.

#### Motivation and Purpose

Describing a clearly stated motivation for using a game over a traditional assessment can justify the use of games in this context. For example, motivation may be to increase engagement with children or reduce dropout rates for long-term monitoring. The game can then be evaluated to determine if it meets the motivation (eg, does it engage children? Does this reduce dropout rates?). This evaluation is important because the field is new enough that the results are often not generalizable.

Clearly articulating a clear long-term goal can set guideposts for the development and evaluation of games for assessment. For example, if the goal is to use a game as a neuropsychology tool, researchers can focus their efforts on making a standardized game that meets robust standards for validity and reliability and has a large normative data set [12]. If the goal is to create a game for widespread dissemination for population-level research, then the focus can be on making the game truly engaging and fun to encourage natural use *in-the-wild*. As an example from our review, McKanna et al [32] identified the need for an unobtrusive way to continuously monitor and detect cognitive decline in older adults. This goal guided their development of a computer game that naturally appealed to older adults and targeted divided attention, a function associated with daily activities that often declines with age.

#### Evaluation

Evaluating the assessment capacity of assessment games is necessary to establish their validity. Given the new variables that any game element introduces to an assessment, moving forward, a focus on robust evaluation needs to be prioritized for any cognitive assessment game. We can look at cognitive psychology for best practices when evaluating tasks. For example, when developing a new parametric go/no-go task, Langenecker et al [106] measured the sensitivity, construct validity, and test-retest reliability. From our review, Chesham et al [50] measured the validity of their search and match task, a puzzle game designed to assess visual search. To do this, they looked at correlation analyses between performance on the game and performance on traditional tasks.

Measuring the player experience within assessment games is necessary to justify the use of games over traditional assessment tasks. In our review, we identified three approaches to evaluating assessment games: comparison to a task, comparison to a clinical diagnosis, and comparison to theories of cognition. Comparisons to a task and to theories of cognition can help determine construct validity. A comparison with a clinical diagnosis may measure the sensitivity. However, there are many other issues that need to be addressed. For example, does the game work for people from different cultures or with different educational backgrounds? Are there practice effects that may interfere with repeated use? Some of the studies in our review addressed questions such as these; for example, Rello et al [94] assessed their game for both English and Spanish speakers; however, every study in our review answered different evaluation questions.

It may be useful to develop best practices around how and what should be evaluated when developing an assessment game. This list should include evaluating the assessment aspects in ways similar to cognitive psychology methods, but it also needs to include evaluating the game aspects. Knowledge on how to evaluate games can come from games user research.

#### Interdisciplinary Work

Integrating knowledge across the disciplines of psychology, clinical sciences, game design, or user experience will help ensure robust results. Regardless of the goals of the assessment game, the best result will often come from an interdisciplinary team. Levy et al [104] noted that, “...the design of scientifically robust games is often at odds with accepted game design practices.” We need to draw from knowledge of both game design and cognitive testing. Interdisciplinary work will be key in developing robust, enjoyable games, and it will also be useful in knowing how to evaluate these games, as discussed earlier. Experts in cognitive psychology, neuropsychology, game design, and games user research can all contribute to this field. As an example from our review, *Smart Aging* by Bottiroli et al [83], a game platform designed to measure various cognitive functions, was developed in collaboration with “neurologists, psychologists, neuropsychologists, bioinformatics, designers, and ICT engineers.”

### Limitations

There are some limitations to our systematic review. We did not search every database for papers; for example, we ruled out using Springer Link because we could not search by title and abstract. We selected our databases based on the relevance to the field and focused on using a mix of computer science– and psychology-related databases. Similarly, we attempted several combinations of search terms. Some yielded too many results for a feasible review. We aimed to search as comprehensively as we could realistically manage. We struck an appropriate balance with 49,365 records to review after duplicates were removed.

We also used cross-referencing and Google Scholar to check for additional papers that met our criteria; however, it is still possible that we missed some papers.

Our review also only addressed papers that used games to assess cognitive processes related to attention to keep this review to a manageable scope. Many other studies have assessed memory and other cognitive functions using games. We intend to cover memory in another review, and future work should cover games that are used to assess more complicated cognitive functions, such as decision-making.

Another limitation of our selection criteria is our reliance on authors to define their work as a game or task. Our search criteria depended on the inclusion of the word *game* in the title or abstract of the paper. We may have missed papers in our review those that used what authors defined as a *task* but still implemented game elements, such as points. In addition, we may have included papers that used what authors defined as a *game* but could be considered a task.

Finally, our review only covers published work and thus, carries the risk of publication bias. As our review focused on the methodologies used by studies and not the outcomes, this issue is not a significant concern.

### Conclusions

We conducted a systematic review to answer the following question: How are digital games used for the cognitive assessment of attention made and measured? We searched a wide range of databases to identify an initial set of 49,365 papers, which we then narrowed to a set of 74 papers that we reviewed in detail. From these studies, we identified three unique approaches for developing a game for assessment. We also identified that, across the field, the focus tends to be on development rather than evaluation. Assumptions about how the application of games to cognitive tasks should improve assessment are widespread but perhaps not widely demonstrated. Our review can act as a resource to help guide the field toward more standardized approaches and rigorous evaluation required for the widespread adoption of assessment games.

## Appendix

Multimedia Appendix 1 General details of each study.

Multimedia Appendix 2 Assessment and game details of each study.

## Abbreviations

ADHD: attention-deficit/hyperactivity disorder
MOT: multiple object tracking
NASA: National Aeronautics and Space Administration

## References

[1] Kahneman D (1973). Attention and Effort.

[2] Wickens C, McCarley J (2008). Applied Attention Theory.

[3] Posner MI, Petersen SE (1990). The attention system of the human brain. Annu Rev Neurosci.

[4] Eysenck MW, Derakshan N (2011). New perspectives in attentional control theory. Pers Individ Differ.

[5] Barkley RA (1997). Behavioral inhibition, sustained attention, and executive functions: constructing a unifying theory of ADHD. Psychol Bull.

[6] Perry RJ, Hodges JR (1999). Attention and executive deficits in Alzheimer's disease. A critical review. Brain.

[7] Franceschini S, Gori S, Ruffino M, Pedrolli K, Facoetti A (2012). A causal link between visual spatial attention and reading acquisition. Curr Biol.

[8] Eriksen BA, Eriksen CW (1974). Effects of noise letters upon the identification of a target letter in a nonsearch task. Percept Psychophys.

[9] Posner MI (1980). Orienting of attention. Q J Exp Psychol.

[10] Bantin T, Stevens S, Gerlach AL, Hermann C (2016). What does the facial dot-probe task tell us about attentional processes in social anxiety? A systematic review. J Behav Ther Exp Psychiatry.

[11] Dux PE, Marois R (2009). The attentional blink: a review of data and theory. Atten Percept Psychophys.

[12] Kessels RP (2019). Improving precision in neuropsychological assessment: bridging the gap between classic paper-and-pencil tests and paradigms from cognitive neuroscience. Clin Neuropsychol.

[13] Lumsden J, Skinner A, Coyle D, Lawrence N, Munafo M (2017). Attrition from web-based cognitive testing: a repeated measures comparison of gamification techniques. J Med Internet Res.

[14] DeRight J, Jorgensen RS (2015). I just want my research credit: frequency of suboptimal effort in a non-clinical healthy undergraduate sample. Clin Neuropsychol.

[15] Kirkwood MW, Kirk JW, Blaha RZ, Wilson P (2010). Noncredible effort during pediatric neuropsychological exam: a case series and literature review. Child Neuropsychol.

[16] Howieson D (2019). Current limitations of neuropsychological tests and assessment procedures. Clin Neuropsychol.

[17] Wiley K, Vedress S, Mandryk R (2020). How points and theme affect performance and experience in a gamified cognitive task. Proceedings of the 2020 CHI Conference on Human Factors in Computing Systems.

[18] Morgan J (2016). Gaming for dementia research: a quest to save the brain. Lancet Neurol.

[19] Abeele VV, Wouters J, Ghesquière P, Goeleven A, Geurts L (2015). Game-based assessment of psycho-acoustic thresholds: not all games are equal!. Proceedings of the 2015 Annual Symposium on Computer-Human Interaction in Play.

[20] Price RB, Kuckertz JM, Siegle GJ, Ladouceur CD, Silk JS, Ryan ND, Dahl RE, Amir N (2015). Empirical recommendations for improving the stability of the dot-probe task in clinical research. Psychol Assess.

[21] Bezdek MA, Gerrig RJ, Wenzel WG, Shin J, Pirog Revill K, Schumacher EH (2015). Neural evidence that suspense narrows attentional focus. Neuroscience.

[22] Delisle J, Braun CM (2011). A context for normalizing impulsiveness at work for adults with attention deficit/hyperactivity disorder (combined type). Arch Clin Neuropsychol.

[23] Chisholm JD, Kingstone A (2015). Action video game players' visual search advantage extends to biologically relevant stimuli. Acta Psychol (Amst).

[24] Deterding S, Dixon D, Khaled R, Nacke L (2011). From game design elements to gamefulness: defining "gamification". Proceedings of the 15th International Academic MindTrek Conference: Envisioning Future Media Environments.

[25] Lumsden J, Edwards EA, Lawrence NS, Coyle D, Munafò MR (2016). Gamification of cognitive assessment and cognitive training: a systematic review of applications and efficacy. JMIR Serious Games.

[26] Vermeir JF, White MJ, Johnson D, Crombez G, Van Ryckeghem DM (2020). The effects of gamification on computerized cognitive training: systematic review and meta-analysis. JMIR Serious Games.

[27] Mandryk RL, Birk MV (2017). Toward game-based digital mental health interventions: player habits and preferences. J Med Internet Res.

[28] Ouzzani M, Hammady H, Fedorowicz Z, Elmagarmid A (2016). Rayyan-a web and mobile app for systematic reviews. Syst Rev.

[29] Mukherjee D, Bhavnani S, Swaminathan A, Verma D, Parameshwaran D, Divan G, Dasgupta J, Sharma K, Thiagarajan TC, Patel V (2020). Proof of concept of a gamified DEvelopmental Assessment on an E-Platform (DEEP) tool to measure cognitive development in rural Indian preschool children. Front Psychol.

[30] Mash LE, Klein RM, Townsend J (2020). Brief report: a gaming approach to the assessment of attention networks in autism spectrum disorder and typical development. J Autism Dev Disord.

[31] Boujut A, Mellah S, Lussier M, Maltezos S, Verty LV, Bherer L, Belleville S (2020). Assessing the effect of training on the cognition and brain of older adults: protocol for a three-arm randomized double-blind controlled trial (ACTOP). JMIR Res Protoc.

[32] McKanna JA, Jimison H, Pavel M (2009). Divided attention in computer game play: analysis utilizing unobtrusive health monitoring. Annu Int Conf IEEE Eng Med Biol Soc.

[33] Mwamba HM, Fourie PR, den Heever DV (2019). PANDAS: Paediatric attention-deficit/hyperactivity disorder application software. Annu Int Conf IEEE Eng Med Biol Soc.

[34] Peijnenborgh JC, Hurks PP, Aldenkamp AP, van der Spek ED, Rauterberg GWM, Vles JS, Hendriksen JG (2016). A study on the validity of a computer-based game to assess cognitive processes, reward mechanisms, and time perception in children aged 4-8 years. JMIR Serious Games.

[35] Rello L, Romero E, Rauschenberger M, Ali A, Williams K, Bigham J, White N (2018). Screening dyslexia for English using HCI measures and machine learning. Proceedings of the 2018 International Conference on Digital Health.

[36] Ryokai K, Farzin F, Kaltman E, Niemeyer G (2013). Assessing multiple object tracking in young children using a game. Edu Tech Res Dev.

[37] Szalma JL, Schmidt TN, Teo GW, Hancock PA (2014). Vigilance on the move: video game-based measurement of sustained attention. Ergonomics.

[38] Thirkettle M, Lewis J, Langdridge D, Pike G (2018). A mobile app delivering a gamified battery of cognitive tests designed for repeated play (OU Brainwave): app design and cohort study. JMIR Serious Games.

[39] Tong T, Chignell M (2014). Developing a serious game for cognitive assessment: choosing settings and measuring performance. Proceedings of the Second International Symposium of Chinese CHI.

[40] Tong T, Chignell M, Tierney MC, Lee J (2016). A serious game for clinical assessment of cognitive status: validation study. JMIR Serious Games.

[41] Wu J, Wang S, Cheng K, Chien P, Lhotska L, Sukupova L, Lacković I, Ibbott G (2019). Computerized cognitive assessment system for dementia screening application. World Congress on Medical Physics and Biomedical Engineering 2018.

[42] Berger A, Jones L, Rothbart MK, Posner MI (2000). Computerized games to study the development of attention in childhood. Behav Res Methods Instrum Comput.

[43] Friehs MA, Dechant M, Vedress S, Frings C, Mandryk RL (2020). Effective gamification of the stop-signal task: two controlled laboratory experiments. JMIR Serious Games.

[44] Apiquian R, Ulloa RE, Victoria G, Gómez-Tello MF, Morales E, García-Covarrubias L (2020). Standardization and validity of Chefmania, a video game designed as a cognitive screening test for children. Humanit Soc Sci Commun.

[45] Hsu W, Rowles W, Anguera JA, Zhao C, Anderson A, Alexander A, Sacco S, Henry R, Gazzaley A, Bove R (2021). Correction: Application of an adaptive, digital, game-based approach for cognitive assessment in multiple sclerosis: observational study. J Med Internet Res.

[46] Filho MD, Boato E, Quesada A, Moresi E, Tristão R (2020). Evaluation of executive functions of children with down syndrome and zika virus using touch screen device?: cognitive evaluation of toddlers by touch-screen. Preoceedings of the 11th IEEE International Conference on Cognitive Infocommunications (CogInfoCom).

[47] Tlili A, Najjar R, Essalmi F, Jemni M, Chang M, Huang R, Chang TW (2020). Unobtrusive monitoring of learners’ game interactions to identify their dyslexia level. Proceedings of the IEEE 20th International Conference on Advanced Learning Technologies (ICALT).

[48] Song H, Yi D, Park H (2020). Validation of a mobile game-based assessment of cognitive control among children and adolescents. PLoS One.

[49] Brown HR, Zeidman P, Smittenaar P, Adams RA, McNab F, Rutledge RB, Dolan RJ (2014). Crowdsourcing for cognitive science - the utility of smartphones. PLoS One.

[50] Chesham A, Gerber SM, Schütz N, Saner H, Gutbrod K, Müri RM, Nef T, Urwyler P (2019). Search and match task: development of a taskified match-3 puzzle game to assess and practice visual search. JMIR Serious Games.

[51] Giglioli IA, de Juan Ripoll C, Parra E, Raya MA (2018). EXPANSE: A novel narrative serious game for the behavioral assessment of cognitive abilities. PLoS One.

[52] Houghton S, Milner N, West J, Douglas G, Lawrence V, Whiting K, Tannock R, Durkin K (2004). Motor control and sequencing of boys with Attention-Deficit/Hyperactivity Disorder (ADHD) during computer game play. Br J Educ Technol.

[53] Jung H, Daneault J, Lee H, Kim K, Kim B, Park S, Ryu T, Kim Y, Lee SI (2019). Remote assessment of cognitive impairment level based on serious mobile game performance: an initial proof of concept. IEEE J Biomed Health Inform.

[54] Jung H, Lee H, Kim K, Kim B, Park S, Ryu T (2018). Estimating mini mental state examination scores using game-specific performance values: a preliminary study. 2018 40th Annu Int Conf IEEE Eng Med Biol Soc.

[55] Ludovico LA, Di Tore PA, Mangione GR, Di Tore S, Corona F (2015). Measuring the reading abilities of dyslexic children through a visual game. Int J Emerg Technol Learn.

[56] Anguera JA, Brandes-Aitken AN, Rolle CE, Skinner SN, Desai SS, Bower JD, Martucci WE, Chung WK, Sherr EH, Marco EJ (2016). Characterizing cognitive control abilities in children with 16p11.2 deletion using adaptive 'video game' technology: a pilot study. Transl Psychiatry.

[57] Baniqued PL, Lee H, Voss MW, Basak C, Cosman JD, Desouza S, Severson J, Salthouse TA, Kramer AF (2013). Selling points: What cognitive abilities are tapped by casual video games?. Acta Psychol (Amst).

[58] Irwin-Chase H, Burns B (2000). Developmental changes in children's abilities to share and allocate attention in a dual task. J Exp Child Psychol.

[59] Johann VE, Karbach J (2018). Validation of new online game-based executive function tasks for children. J Exp Child Psychol.

[60] Kawahara Y, Ikeda Y, Deguchi K, Kurata T, Hishikawa N, Sato K, Kono S, Yunoki T, Omote Y, Yamashita T, Abe K (2015). Simultaneous assessment of cognitive and affective functions in multiple system atrophy and cortical cerebellar atrophy in relation to computerized touch-panel screening tests. J Neurol Sci.

[61] Loleski M, Loleska S, Pop-Jordanova N (2017). Mobile application "Neurogame" for assessment the attention, focus and concentration. Pril (Makedon Akad Nauk Umet Odd Med Nauki).

[62] Lumsden J, Skinner A, Woods AT, Lawrence NS, Munafò M (2016). The effects of gamelike features and test location on cognitive test performance and participant enjoyment. PeerJ.

[63] Michel JA, Kerns KA, Mateer CA (2005). The effect of reinforcement variables on inhibition in children with ADHD. Child Neuropsychol.

[64] Miranda AT, Palmer EM (2014). Intrinsic motivation and attentional capture from gamelike features in a visual search task. Behav Res Methods.

[65] Pereira VF, Valentin LS (2018). The MentalPlus® digital game might be an accessible open source tool to evaluate cognitive dysfunction in heart failure with preserved ejection fraction in hypertensive patients: a pilot exploratory study. Int J Hypertens.

[66] Shaw R, Grayson A, Lewis V (2005). Inhibition, ADHD, and computer games: the inhibitory performance of children with ADHD on computerized tasks and games. J Atten Disord.

[67] Delgado MT, Uribe PA, Alonso AA, Díaz RR (2016). TENI: A comprehensive battery for cognitive assessment based on games and technology. Child Neuropsychol.

[68] Bonnechère B, Fabris C, Bier J, Van Sint Jan S, Feipel V, Jansen B (2016). Evaluation of cognitive functions of aged patients using video games. Proceedings of the 4th Workshop on ICTs for Improving Patients Rehabilitation Research Techniques.

[69] Tong T, Chignell M, DeGuzman CA (2019). Using a serious game to measure executive functioning: response inhibition ability. Appl Neuropsychol Adult.

[70] Tong T, Chignell M, Tierney MC, Lee JS (2016). Test-retest reliability of a serious game for delirium screening in the emergency department. Front Aging Neurosci.

[71] Tulloch K, Pammer K (2019). Tablet computer games to measure dorsal stream performance in good and poor readers. Neuropsychologia.

[72] Valladares-Rodriguez S, Fernández-Iglesias MJ, Anido-Rifón L, Facal D, Rivas-Costa C, Pérez-Rodríguez R (2019). Touchscreen games to detect cognitive impairment in senior adults. A user-interaction pilot study. Int J Med Inform.

[73] van Hemel-Ruiter ME, de Jong PJ, Oldehinkel AJ, Ostafin BD (2013). Reward-related attentional biases and adolescent substance use: the TRAILS study. Psychol Addict Behav.

[74] Van Hove O, Van Muylem A, Andrianopoulos V, Leduc D, Feipel V, Deboeck G, Bonnechère B (2020). The use of cognitive mobile games to assess the interaction of cognitive function and breath-hold. Respir Physiol Neurobiol.

[75] Wilson BJ, Petaja H, Mancil L (2011). The attention skills and academic performance of aggressive/rejected and low aggressive/popular children. Early Educ Dev.

[76] Wisniewski H, Henson P, Torous J (2019). Using a smartphone app to identify clinically relevant behavior trends symptom report, cognition scores, and exercise levels: a case series. Front Psychiatry.

[77] Berg V, Rogers SL, McMahon M, Garrett M, Manley D (2020). A novel approach to measure executive functions in students: an evaluation of two child-friendly apps. Front Psychol.

[78] Delgado-Gómez D, Sújar A, Ardoy-Cuadros J, Bejarano-Gómez A, Aguado D, Miguelez-Fernandez C, Blasco-Fontecilla H, Peñuelas-Calvo I (2020). Objective Assessment of Attention-Deficit Hyperactivity Disorder (ADHD) using an infinite runner-based computer game: a pilot study. Brain Sci.

[79] Bonnechère B, Van Vooren M, Bier J, De Breucker S, Van Hove O, Van Sint Jan S, Feipel V, Jansen B (2018). The use of mobile games to assess cognitive function of elderly with and without cognitive impairment. J Alzheimers Dis.

[80] Chen Y, Hou C, Derek N, Huang S, Huang M, Wang Y (2021). Evaluation of the reaction time and accuracy rate in normal subjects, MCI, and dementia using serious games. Appl Sci.

[81] Nef T, Chesham A, Schütz N, Botros AA, Vanbellingen T, Burgunder J, Müllner J, Müri RM, Urwyler P (2020). Development and evaluation of maze-like puzzle games to assess cognitive and motor function in aging and neurodegenerative diseases. Front Aging Neurosci.

[82] Gómez-Tello MF, Rosetti MF, Galicia-Alvarado M, Maya C, Apiquian R (2020). Neuropsychological screening with TOWI: Performance in 6- to 12-year-old children. Appl Neuropsychol Child.

[83] Bottiroli S, Tassorelli C, Lamonica M, Zucchella C, Cavallini E, Bernini S, Sinforiani E, Pazzi S, Cristiani P, Vecchi T, Tost D, Sandrini G (2017). Smart aging platform for evaluating cognitive functions in aging: a comparison with the MoCA in a normal population. Front Aging Neurosci.

[84] Colonna A, Smith AB, Smith S, VanDenEshof K, Orgill J, Gringras P, Pal DK (2018). The effects of sleep on emotional target detection performance: a novel iPad-based pediatric game. Front Psychol.

[85] Dibbets P, Jolles J (2006). The Switch Task for Children: Measuring mental flexibility in young children. Cogn Dev.

[86] Dunbar G, Hill R, Lewis V (2001). Children's attentional skills and road behavior. J Exp Psychol Appl.

[87] Flynn RM, Colón-Acosta N, Zhou J, Bower J (2019). A game-based repeated assessment for cognitive monitoring: initial usability and adherence study in a summer camp setting. J Autism Dev Disord.

[88] Fukui Y, Yamashita T, Hishikawa N, Kurata T, Sato K, Omote Y, Kono S, Yunoki T, Kawahara Y, Hatanaka N, Tokuchi R, Deguchi K, Abe K (2015). Computerized touch-panel screening tests for detecting mild cognitive impairment and Alzheimer's disease. Intern Med.

[89] Gaggi O, Galiazzo G, Palazzi C, Facoetti A, Franceschini S (2012). A serious game for predicting the risk of developmental dyslexia in pre-readers children. 21st International Conference on Computer Communications and Networks (ICCCN).

[90] Godwin K, Lomas D, Koedinger K, Fisher AV (2015). Monster Mischief: Designing a video game to assess selective sustained attention. Int J Gaming Comput Mediat Simul.

[91] Valladares-Rodríguez S, Anido-Rifón L, Fernández-Iglesias M, Facal-Mayo D, Misra S, Gervasi O, Murgante B, Stankova E, Korkhov V, Torre C, Rocha A, Taniar D, Apduhan B, Tarantino E (2019). A machine learning approach to the early diagnosis of Alzheimer’s disease based on an ensemble of classifiers. Computational Science and Its Applications – ICCSA 2019.

[92] Intarasirisawat J, Ang CS, Efstratiou C, Dickens LW, Page R (2019). Exploring the touch and motion features in game-based cognitive assessments. Proceedings of the ACM on Interactive, Mobile, Wearable and Ubiquitous Technologies.

[93] Waters L, Blackmore K, van der Spek E, Göbel S, Do EY, Clua E, Hauge JB (2019). Using game-based environments to measure cognitive decision making. Entertainment Computing and Serious Games.

[94] Rello L, Williams K, Ali A, White N, Bigham J (2016). Dytective: towards detecting dyslexia across languages using an online game. Proceedings of the 13th International Web for All Conference.

[95] Rosetti MF, Gómez-Tello MF, Victoria G, Apiquian R (2017). A video game for the neuropsychological screening of children. Entertain Comput.

[96] Gaggi O, Palazzi CE, Ciman M, Galiazzo G, Franceschini S, Ruffino M, Gori S, Facoetti A (2017). Serious games for early identification of developmental dyslexia. Comput Entertain.

[97] Silva Neto H, Cerejeira J, Roque L (2018). Cognitive screening of older adults using serious games: an empirical study. Entertain Comput.

[98] Wallace B, Knoefel F, Goubran R, Masson P, Baker A, Allard B, Guana V, Stroulia E (2018). Detecting cognitive ability changes in patients with moderate dementia using a modified “Whack-a-Mole” game. IEEE Trans Instrum Meas.

[99] Li B, Atyabi A, Kim M, Barney E, Ahn A, Luo Y, Aubertine M, Corrigan S, John T, Wang Q, Mademtzi M, Best M, Shic F (2018). Social influences on executive functioning in autism: design of a mobile gaming platform. Proceedings of the 2018 CHI Conference on Human Factors in Computing Systems.

[100] Rauschenberger M, Rello L, Baeza-Yates R, Bigham J (2018). Towards language independent detection of dyslexia with a web-based game. Proceedings of the 15th International Web for All Conference.

[101] Zeng Z, Fauvel S, Hsiang B, Wang D, Qiu Y, Khuan P, Leung C, Shen Z, Chin JJ (2018). Towards long-term tracking and detection of early dementia: a computerized cognitive test battery with gamification. Proceedings of the 3rd International Conference on Crowd Science and Engineering.

[102] Veenstra B, van Geert PL, van der Meulen BF (2012). Distinguishing and improving mouse behavior with educational computer games in young children with autistic spectrum disorder or attention deficit/hyperactivity disorder: an executive function-based interpretation. Mind, Brain, Educ.

[103] Arizmendi GD, Alt M, Gray S, Hogan TP, Green S, Cowan N (2018). Do bilingual children have an executive function advantage? Results from inhibition, shifting, and updating tasks. Lang Speech Hear Serv Sch.

[104] Levy L, Lambeth A, Solomon R, Gandy M (2018). Method in the madness: the design of games as valid and reliable scientific tools. Proceedings of the 13th International Conference on the Foundations of Digital Games.

[105] Fullerton T (2014). Game Design Workshop: A Playcentric Approach to Creating Innovative Games, Third Edition.

[106] Langenecker SA, Zubieta J, Young EA, Akil H, Nielson KA (2007). A task to manipulate attentional load, set-shifting, and inhibitory control: convergent validity and test-retest reliability of the Parametric Go/No-Go Test. J Clin Exp Neuropsychol.

