# Supplementary material for: The Making and Evaluation of Digital Games Used for the Assessment of Attention: Systematic Review
Source: JMIR Serious Games. 2021 Aug 9;9(3):e26449. doi: 10.2196/26449 (PMC8386381; doi:10.2196/26449)
Supplement: Multimedia Appendix 1 [file games_v9i3e26449_app1.docx]

## Appendix 1. General details of each study

| **Reference** | **Year** | **Field of Journal** | **Sample Size** | **Area of Attention** | **Population Age** | **Disorder** |
| --- | --- | --- | --- | --- | --- | --- |
|  |  | *1 = interdisciplinary*  *2 = psych/medical*  *3 = computer science*  *4 = education* |  | *1 = visual*  *2 = divided*  *3 = selective*  *4 = sustained*  *5 = inhibition*  *6 = switching*  *7 = updating*  *8 = orienting*  *9 = general/not specified*  *10 = attentional bias* | *1 = children*  *2 = older adults*  *3 = general adults* | *1 = ADHD and ASD*  *2 = dyslexia*  *3 = dementia and cognitive impairment*  *4 = other*  *5 = none* |
| 13 | 2017 | 1 | 291 | 5 | 3 | 5 |
| 17 | 2020 | 1 | 740 | 10 | 3 | 5 |
| 32 | 2009 | 1 | 30 | 2 | 2 | 5 |
| 33 | 2019 | 1 | 30 | 4 | 1 | 1 |
| 34 | 2016 | 1 | 136 | 4, 5 | 1 | 1 |
| 35 | 2018 | 1 | 267 | 1 | 1, 3 | 2 |
| 36 | 2013 | 1 | 31 | 1 | 1 | 5 |
| 37 | 2014 | 1 | 28 | 4 | 3 | 5 |
| 38 | 2018 | 1 | 13979 | 2, 4 | 3 | 5 |
| 39 | 2014 | 1 | 24 | 5 | 3 | 5 |
| 40 | 2016 | 1 | 141 | 5 | 2 | 3 |
| 41 | 2019 | 1 | 10 | 9 | 3 | 3 |
| 42 | 2000 | 1 | 30 | 8 | 1 | 5 |
| 43 | 2020 | 1 | 30 | 5 | 3 | 5 |
| 44 | 2020 | 1 | 266 | 3, 5 | 1 | 1 |
| 45 | 2021 | 1 | 100 | 3 | 3 | 4 |
| 46 | 2020 | 1 | 107 | 4 | 1 | 4 |
| 47 | 2020 | 1 | 26 | 1 | 1 | 2 |
| 48 | 2020 | 1 | 100 | 4, 5 | 1 | 5 |
| 49 | 2014 | 1 | 16233 | 3 | 3 | 5 |
| 50 | 2019 | 1 | 52 | 1 | 1, 2, 3 | 5 |
| 51 | 2018 | 1 | 354 | 5, 6 | 3 | 5 |
| 52 | 2004 | 1 | 146 | 4 | 1 | 1 |
| 53 | 2019 | 1 | 12 | 1, 3 | 3 | 3 |
| 54 | 2018 | 1 | 12 | 3 | 3 | 3 |
| 55 | 2015 | 1 | 12 | 1 | 1 | 2 |
| 56 | 2016 | 2 | 111 | 3 | 1 | 1 |
| 57 | 2013 | 2 | 209 | 2 | 3 | 5 |
| 58 | 2000 | 2 | 20 | 2 | 1 | 5 |
| 59 | 2018 | 2 | 60 | 5 | 1 | 5 |
| 60 | 2015 | 2 | 153 | 1 | 2 | 3 |
| 61 | 2017 | 2 | 201 | 4 | 3 | 5 |
| 62 | 2016 | 2 | 100 | 5 | 3 | 5 |
| 63 | 2005 | 2 | 40 | 5 | 1 | 1 |
| 64 | 2014 | 2 | 24 | 1 | 3 | 5 |
| 65 | 2018 | 2 | 60 | 3, 5 | 3 | 3 |
| 66 | 2005 | 2 | 32 | 4 | 1 | 1 |
| 67 | 2016 | 2 | 524 | 4 | 1 | 5 |
| 68 | 2016 | 2 | 20 | 1, 4, 5, 6 | 3 | 3 |
| 69 | 2019 | 2 | 30 | 5 | 3 | 5 |
| 70 | 2016 | 2 | 114 | 4, 5 | 2 | 3 |
| 71 | 2019 | 2 | 83 | 1 | 1 | 5 |
| 72 | 2019 | 2 | 75 | 9 | 2 | 3 |
| 73 | 2013 | 2 | 682 | 8 | 1 | 4 |
| 74 | 2019 | 2 | 31 | 4, 5, 6 | 3 | 5 |
| 75 | 2011 | 2 | 54 | 4 | 1 | 4 |
| 76 | 2019 | 2 | 5 | 1 | 3 | 4 |
| 77 | 2020 | 2 | 81 | 5 | 1 | 5 |
| 78 | 2020 | 2 | 32 | 9 | 1 | 1 |
| 79 | 2018 | 2 | 76 | 1, 4, 5, 6 | 3 | 3 |
| 80 | 2021 | 2 | 37 | 5 | 2 | 3 |
| 81 | 2020 | 2 | 55 | 1, 5, 6 | 3 | 4 |
| 82 | 2020 | 2 | 1139 | 3, 4, 5 | 1 | 5 |
| 83 | 2017 | 2 | 1086 | 2, 3 | 2 | 5 |
| 84 | 2018 | 2 | 25 | 3, 4 | 1 | 5 |
| 85 | 2006 | 2 | 292 | 6 | 1 | 5 |
| 86 | 2001 | 2 | 160 | 6 | 1 | 5 |
| 87 | 2019 | 2 | 130 | 3, 4 | 1 | 1 |
| 88 | 2015 | 2 | 240 | 1 | 2 | 3 |
| 89 | 2012 | 3 | 24 | 1 | 1 | 2 |
| 90 | 2015 | 3 | 27 | 3, 4 | 1 | 5 |
| 91 | 2019 | 3 | 64 | 9 | 2 | 3 |
| 92 | 2019 | 3 | 22 | 1, 4 | 3 | 5 |
| 93 | 2019 | 3 | 41 | 2 | 3 | 5 |
| 94 | 2016 | 3 | 60 | 1 | 1 | 2 |
| 95 | 2017 | 3 | 75 | 3, 4, 5 | 1 | 5 |
| 96 | 2017 | 3 | 24 | 1 | 1 | 2 |
| 97 | 2018 | 3 | 103 | 9 | 2 | 3 |
| 98 | 2018 | 3 | 11 | 4, 5 | 2 | 3 |
| 99 | 2018 | 3 | 65 | 5, 6 | 1 | 1 |
| 100 | 2018 | 3 | 178 | 1 | 1 | 2 |
| 101 | 2018 | 3 | 13 | 3, 4 | 2 | 3 |
| 102 | 2012 | 4 | 32 | 5 | 1 | 1 |
| 103 | 2018 | 4 | 247 | 5, 6, 7 | 1 | 5 |
