# Supplementary material for: The Making and Evaluation of Digital Games Used for the Assessment of Attention: Systematic Review
Source: JMIR Serious Games. 2021 Aug 9;9(3):e26449. doi: 10.2196/26449 (PMC8386381; doi:10.2196/26449)
Supplement: Multimedia Appendix 2 [file games_v9i3e26449_app2.docx]

## Appendix 2. Assessment and game details of each study.

| **Reference** | **Assessment Evaluation** | **Purpose of Game** | **Game Development** | **Game Evaluation** |
| --- | --- | --- | --- | --- |
|  | *1 = Task comparison*  *2 = Clinical comparison*  *3 = Theory comparison*  *4 = None* | *1 = ecological validity*  *2 = engage children*  *3 = address limitations of traditional tests*  *4 = increase motivation*  *5 = previous research*  *6 = none* | *1 = gamification*  *2 = custom game based on theory*  *3 = commercial game*  *4 = other* | *1 = yes*  *2 = no evaluation*  *3 = no formal evaluation* |
| 13 | 1 | 4 | 1 | 1 |
| 17 | 1 | 4 | 1 | 1 |
| 32 | 4 | 3 | 2 | 2 |
| 33 | 2 | 3 | 2 | 3 |
| 34 | 2 | 2 | 2 | 2 |
| 35 | 2 | 3 | 2 | 2 |
| 36 | 3 | 2 | 1 | 3 |
| 37 | 3 | 4 | 1 | 1 |
| 38 | 3 | 4 | 1 | 3 |
| 39 | 1 | 3, 4 | 2 | 3 |
| 40 | 1 | 4 | 1 | 1 |
| 41 | 1 | 3, 4 | 2 | 1 |
| 42 | 4 | 2 | 1 | 3 |
| 43 | 1 | 4 | 1 | 1 |
| 44 | 1, 2 | 3 | 1 | 2 |
| 45 | 1,2 | 3 | 2 | 2 |
| 46 | 2, 3 | 2 | 2 | 2 |
| 47 | 2 | 2, 3 | 2 | 1 |
| 48 | 1 | 1, 2, 3 | 1 | 2 |
| 49 | 3 | 4 | 1 | 3 |
| 50 | 1 | 5 | 2 | 1 |
| 51 | 1 | 1 | 1 | 2 |
| 52 | 2 | 1, 2 | 3 | 2 |
| 53 | 1 | 3 | 2 | 1 |
| 54 | 1 | 3 | 2 | 2 |
| 55 | 1 | 5 | 2 | 2 |
| 56 | 1 | 2 | 2 | 2 |
| 57 | 1 | 4 | 3 | 3 |
| 58 | 3 | 2 | 1 | 2 |
| 59 | 1 | 4 | 1 | 1 |
| 60 | 2 | 6 | 2 | 2 |
| 61 | 4 | 6 | 2 | 2 |
| 62 | 1 | 4 | 1 | 1 |
| 63 | 1 | 2 | 1 | 2 |
| 64 | 3 | 4 | 2 | 1 |
| 65 | 1 | 3 | 2 | 2 |
| 66 | 1 | 2 | 1, 3 | 1 |
| 67 | 1 | 4 | 2 | 2 |
| 68 | 1 | 5 | 2 | 2 |
| 69 | 1 | 3, 4 | 2 | 2 |
| 70 | 1 | 5 | 1 | 2 |
| 71 | 3 | 2 | 1 | 2 |
| 72 | 4 | 3 | 1 | 1 |
| 73 | 4 | 5 | 1 | 1 |
| 74 | 4 | 3 | 1 | 2 |
| 75 | 4 | 6 | 2 | 2 |
| 76 | 4 | 5 | 1 | 2 |
| 77 | 3 | 1, 2 | 1 | 1 |
| 78 | 2 | 3 | 2 | 2 |
| 79 | 1 | 6 | 2 | 2 |
| 80 | 2 | 3 | 2 | 2 |
| 81 | 1, 2 | 1, 3, 4 | 2 | 1 |
| 82 | 3 | 1, 2 | 1 | 2 |
| 83 | 1 | 3, 4 | 2 | 2 |
| 84 | 4 | 6 | 1 | 2 |
| 85 | 3 | 2 | 2 | 2 |
| 86 | 4 | 1 | 1 | 2 |
| 87 | 1, 2 | 4 | 2 | 1 |
| 88 | 2 | 6 | 2 | 2 |
| 89 | 4 | 4 | 2 | 1 |
| 90 | 1 | 5 | 1 | 1 |
| 91 | 2 | 3 | 1 | 3 |
| 92 | 1 | 4 | 3 | 2 |
| 93 | 1 | 4 | 1 | 2 |
| 94 | 2 | 2, 3 | 2 | 2 |
| 95 | 1 | 3 | 1 | 2 |
| 96 | 2 | 3 | 2 | 1 |
| 97 | 1 | 3 | 2 | 2 |
| 98 | 1 | 3 | 2 | 1 |
| 99 | 2 | 2, 3 | 1 | 1 |
| 100 | 2 | 2 | 2 | 1 |
| 101 | 4 | 4 | 1 | 1 |
| 102 | 3 | 1 | 2 | 2 |
| 103 | 4 | 6 | 1 | 2 |
